# Endosonography-Guided Tissue Acquisition for Diagnosis of Squamous Carcinoma of the Pancreas: A Report of Three Cases

**DOI:** 10.7759/cureus.31344

**Published:** 2022-11-10

**Authors:** José C Ardengh, Otavio Micelli-Neto, Rafael Kemp, José Sebastião dos Santos

**Affiliations:** 1 Surgery and Anatomy, Hospital das Clinicas, Faculty of Medicine, University of São Paulo, Ribeirão Preto, BRA; 2 Diagnostic Imaging, Universidade Federal de São Paulo, São Paulo, BRA; 3 Digestive Endoscopy Service, Hospital Moriah, São Paulo, BRA

**Keywords:** carcinoma pancreas, tissue viability, endosonography, needle biopsy, adenosquamous carcinoma

## Abstract

While approximately 85% of neoplasms are ductal pancreatic adenocarcinomas (DPA), adenosquamous pancreatic carcinoma (APC) is a rare subtype of pancreatic cancer that exhibits aggressive behavior and poor prognosis. The authors report three cases of primary APC diagnosed through endoscopic ultrasound-guided tissue acquisition (EUS-TA) using the new ProCore 20G needle, which had been developed to improve fine-needle aspiration results by providing more tissue for histopathology. Given its ability for microcore retrieval, pancreatic stroma examination, and excellent histopathology results, EUS-TA has exhibited exceptional diagnostic yield among patients with solid pancreatic lesions. All three APC cases presented herein had been accurately diagnosed using immunohistochemistry after microcore acquisition.

## Introduction

More than 95% of malignant pancreatic tumors arise from exocrine cells. Ductal pancreatic adenocarcinoma (DPA) is the second most common gastrointestinal malignancy and its frequency is approximately 85% of pancreatic neoplasms [1]. Primary adenosquamous pancreatic carcinoma (APC) is a rare subtype of pancreatic cancer that exhibits aggressive behavior with a poor prognosis [2]. Comprising only 1-4% of exocrine pancreatic neoplasms, APC demonstrates squamous and malignant glandular differentiation [3,4].

Endoscopic ultrasound (EUS) guided fine needle aspiration (FNA) is the preferred technique for cell sampling for solid pancreatic lesions, and tissue acquisition (TA) was recently developed to improve the outcome. Endoscopic ultrasound-guided tissue acquisition (EUS-TA) seems to decrease the number of punctures and obtain more tissue, with better specimens of solid pancreatic tumors. Nowadays, the tendency is to use the first-line method in the diagnosis of solid pancreatic lesions [5]. The present case reports describe the role of EUS-TA in the diagnosis, as well as describe the main histopathology findings of the microcore biopsy obtained.

## Case presentation

Case 1

**Figure 1:**
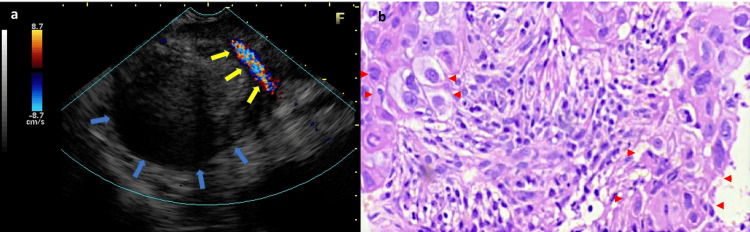
Endoscopic ultrasound and histopathology of Case 1. The images show (a) sectorial scanning revealing a 3.8×4.4 cm, heterogeneous, hypoechoic lesion with an indistinct border (blue arrows) in the body and tail with splenic vein invasion and close contact with the splenic artery (yellow arrows). (b) EUS-TA (microcore) with a solid squamous area (red points) with intense nuclear pleomorphism and desmoplasia (H&E, 100x). EUS-TA: endoscopic ultrasound-guided tissue acquisition

A 46-year-old female presented with a history of thoracolumbar pain for one month. Her computed tomography (CT) and magnetic resonance imaging (MRI) revealed a mass at the pancreatic tail. The lesion measured 4.0×3.7 cm and was in close proximity to the posterior gastric wall. Moreover, no evidence of main pancreatic duct (MPD) dilation and abdominal cavity lymph node enlargement was observed. Endoscopic ultrasound (EUS) revealed a 3.8×4.4 cm, heterogeneous, hypoechoic lesion located in the body and tail with splenic vein invasion and close contact with the splenic artery (Figure 1, panel a). EUS-guided tissue acquisition (EUS-TA) was then performed using the new 20G fine needle biopsy (ProCore 20G; Cook Medical, Bloomington, IN). Accordingly, no lymph nodes peripheral to the tumor were observed. Histopathology shows a sudden change from the glandular/ductal pattern to the squamous component, with polygonal, dyskeratotic cells in a solid neoplastic arrangement (Figure 1, panel b). Adjuvant treatment with gemcitabine was thereafter initiated. However, the disease continued to progress resulting in the patient’s death.

Case 2

**Figure 2:**
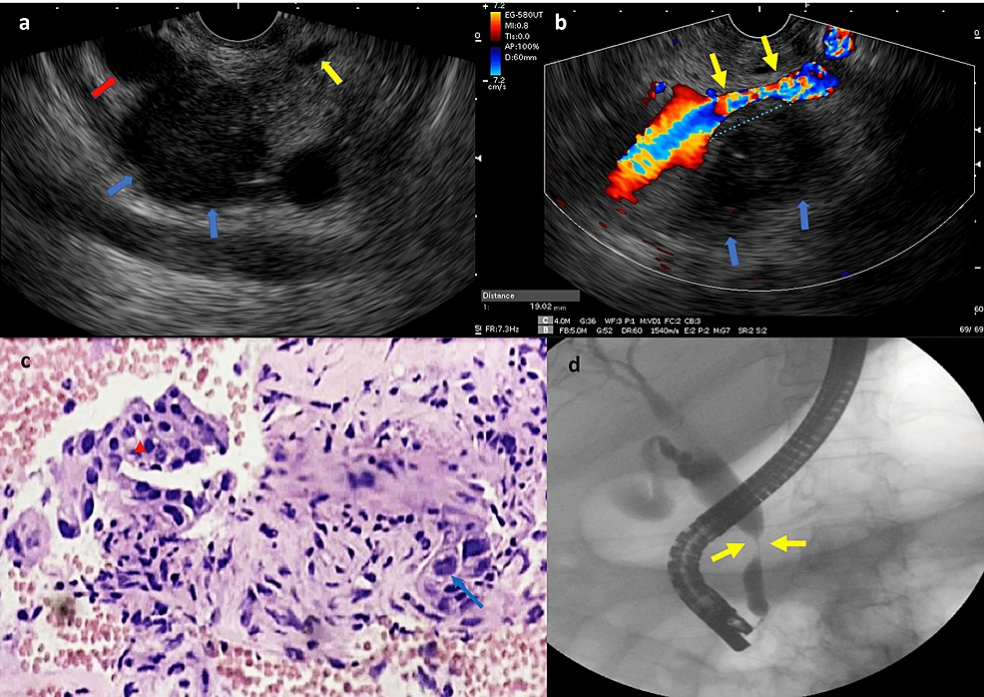
Endoscopic ultrasound, EUS-TA, histopathology, and ERCP of Case 2. The images show (a) EUS revealing a heterogeneous, hypoechoic lesion with an indistinct border in the uncinate process and pancreatic head (blue arrows). Common bile duct (red arrow) and main pancreatic duct (yellow arrow) involvement was observed. (b) Solid and hypoechoic lesion (blue arrows) located in the head with intimate contact with the superior mesenteric vein (yellow arrows) over a length of 1.9 cm without invasion. (c) Solid microtubular area (redpoint) with squamous differentiation (blue arrow) and desmoplastic stroma can be seen (H&E, 100x). (d) ERCP showed a stenosis of the CBD in the head of the pancreas (yellow arrows) treated at the same time of EUS-TA with a biliary self-expandable metallic stent. EUS: endoscopic ultrasound, EUS-TA: endoscopic ultrasound-guided tissue acquisition; ERCP: endoscopic retrograde cholangiopancreatography, CBD: common bile duct

A 69-year-old female presented with abdominal pain that radiated to the back. Her contrast-enhanced (CT) showed an infiltrative lesion in the uncinate process and pancreatic head leading to choledochal dilatation without MPD dilation. The patient was scheduled for EUS-TA and endoscopic retrograde cholangiopancreatography (ERCP) for biliary drainage considering that the common bile duct (CBD) had been compromised. EUS showed a 2.6×3.1 cm, solid, hypoechoic, well-defined lesion located in the head of the pancreas (Figure 2, panel a). CBD and MPD involvement were noted, causing the dilation of the former. The tumor presented intimate contact with the superior mesenteric vein over a length of 1.9 cm without causing invasion (Figure 2, panel b). EUS-TA was then performed using the ProCore 20G needle. Accordingly, microcore biopsy comprised both squamous carcinoma and adenocarcinoma components (Figure 2, panel c). The presence of malignant keratinized squamous cells was the most reliable evidence of squamous differentiation. The patient underwent ERCP (Figure 2, panel d) for biliary drainage using a fully covered metallic stent. Palliative chemotherapy with gemcitabine and nab-paclitaxel was thereafter initiated. The patient has continued to remain under follow-up five months after diagnosis.

Case 3

**Figure 3:**
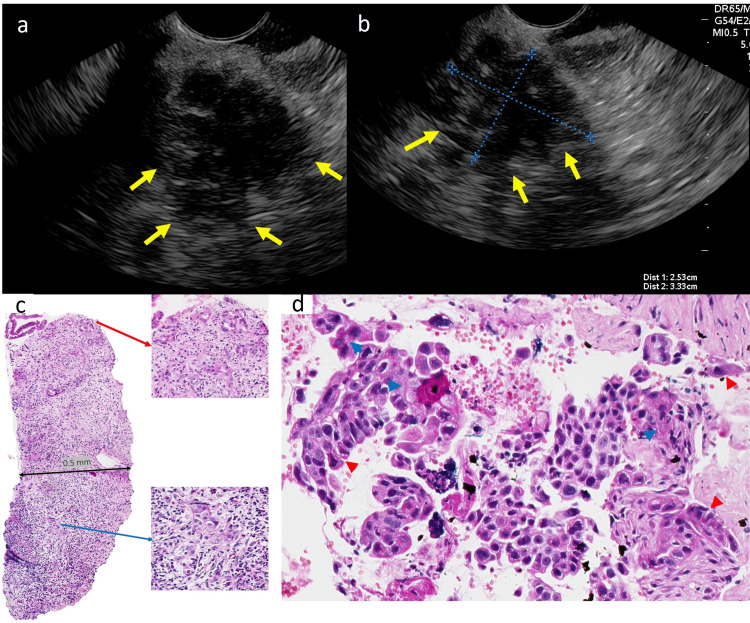
EUS imaging, EUS-TA, and histopathology of Case 3. The images show (a and b) EUS imaging revealing a 3.33×2.53 cm solid, hypoechoic, poorly defined lesion with an irregular contour (yellow arrows). (c) Microcore obtained during EUS-TA in a desmoplastic area with a thickness of 0.5 cm (black arrow), the microtubular component represents the DPA (red arrow), and the differentiation of squamous cells in more than 30% of the specimen shows differentiation into APC (blue arrow). (e) H&E 200x, squamous component with cytoplasmic keratinization (red points) and intercellular bridges (blue points). EUS: endoscopic ultrasound, EUS-TA: endoscopic ultrasound-guided tissue acquisition, DPA: ductal pancreatic adenocarcinomas, APC: adenosquamous pancreatic carcinoma

A 77-year-old male presented with upper abdominal pain that radiated to the back. His contrast-enhanced (CT) showed a 4.2×2.9 cm lesion located in the pancreatic isthmus with MPD dilatation and vascular contact with the splenic-mesenteric confluence and common hepatic and splenic arteries. Moreover, multiple peripherally enhanced nodules scattered throughout the liver parenchyma were observed (Figure 3, panel a). The patient was then scheduled for EUS-TA. EUS showed a 2.5×3.3 cm, solid, hypoechoic lesion with an irregular contour located in the pancreatic isthmus, as well as MPD dilatation (Figure 3, panels b and c). The close vascular contact with the spleno-mesenteric confluence and the splenic and common hepatic arteries was confirmed during the examination. Microcore biopsy revealed the presence of squamous carcinoma components in up to 30% of the material obtained and adenocarcinoma. A week after the procedure, the patient died due to disease severity.

## Discussion

Several types of pancreatic neoplasms have been identified, including adenocarcinoma, adenosquamous pancreatic carcinoma, solid pseudopapillary neoplasm, neuroendocrine neoplasia, squamous cell carcinoma, cystic tumors, primary lymphoma, and metastatic pancreatic lesions [6]. Accordingly, APC, a rare and aggressive subtype of pancreatic cancer, is composed of at least 30% squamous cells in a background of glandular elements [4]. Although most APCs occur in the body and head, they can affect any part of the pancreas as shown in our previously reported case series [7].

After comparing patients with DPA (n=45,693) to those with APC (n=415), Boyd et al. showed that those with APC were more likely to develop the disease at the pancreatic body and tail (44.6% vs. 53.5%, p<0.0001), have poorly differentiated (71% vs. 45%, p<0.0001) and larger (5.7 vs. 4.3 cm, p<0.0001) neoplasms, and have positive lymph nodes (53% vs. 47%, p< 0.0001). After resection, patients with APC had worse survival at two years (29% vs. 36%, p<0.0001) compared to those with DPA [3]. The same occurred in our patients who had disease-free survival of less than one year after diagnosis.

The symptoms and radiological features of APC are nonspecific and indistinguishable from DPA, while its clinical presentation and progression have been based on tumor localization and involvement of adjacent structures [4,8]. Adequate tissue sampling is essential for establishing an accurate diagnosis [9]. Currently, EUS-guided fine-needle aspiration (FNA) has been the gold standard for the diagnosis of pancreatic masses, with a diagnostic accuracy ranging from 77% to 95% [5,10]. Accordingly, a sensitivity of 77% can be achieved by utilizing strict cytological criteria with experienced hands. This low diagnostic rate can be attributed to inadequate specimens, necrosis, and extensive fibrosis [11,12]. Moreover, an absolute majority of cytological samples do not allow for immunohistochemical examination, limiting the cytopathological diagnosis of APC [13].

EUS-TA, which provides more tissue for histology, had been developed to supplant FNA and will do so in the near future given its advantages mainly for solid pancreatic masses, with a sensitivity and specificity of 98.4% and 100%, respectively [14-16]. Notably, this procedure can obtain adequate histological samples (microcore), allowing for the examination of the desmoplastic stroma with diagnostic yield above 94% [9,15].

In the cases presented herein, the diagnosis of APC had been accurately established through the microcore biopsy after performing EUS-TA using the ProCore 20G needle, which allowed for the acquisition of sufficient tissue fragments. An accurate diagnosis is necessary for selecting the appropriate treatment, thereby preventing unnecessary procedures, and improving survival.

## Conclusions

In conclusion, the present report suggests that EUS-TA is a viable alternative for the diagnosis of APC, as it provided sufficient material for histological analysis, and made the correct histological diagnosis, as demonstrated.

## References

[1] Fitzgerald TL, Hickner ZJ, Schmitz M, Kort EJ (2008). Changing incidence of pancreatic neoplasms: a 16-year review of statewide tumor registry. Pancreas.

[2] Na YJ, Shim KN, Cho MS, Sung SH, Jung SA, Yoo K, Chung KW (2011). Primary adenosquamous cell carcinoma of the pancreas: a case report with a review of the Korean literature. Korean J Intern Med.

[3] Boyd CA, Benarroch-Gampel J, Sheffield KM, Cooksley CD, Riall TS (2012). 415 patients with adenosquamous carcinoma of the pancreas: a population-based analysis of prognosis and survival. J Surg Res.

[4] Kelly KC, Moore C (2019). Two rare cases of pancreatic adenosquamous carcinoma: a review of the literature with focus on radiologic findings. Radiol Case Rep.

[5] Conti CB, Cereatti F, Grassia R (2019). Endoscopic ultrasound-guided sampling of solid pancreatic masses: the fine needle aspiration or fine needle biopsy dilemma. Is the best needle yet to come?. World J Gastrointest Endosc.

[6] Cao SH (1992). Extrahepatic bile duct cancer. Report of 106 cases. Chin Med J (Engl).

[7] Schawkat K, Tsai LL, Jaramillo-Cardoso A (2021). Use of ring-enhancement and focal necrosis to differentiate pancreatic adenosquamous carcinoma from pancreatic ductal adenocarcinoma on CT and MRI. Clin Imaging.

[8] Yin Q, Wang C, Wu Z (2013). Adenosquamous carcinoma of the pancreas: multidetector-row computed tomographic manifestations and tumor characteristics. J Comput Assist Tomogr.

[9] Madura JA, Jarman BT, Doherty MG, Yum MN, Howard TJ (1999). Adenosquamous carcinoma of the pancreas. Arch Surg.

[10] Dumonceau JM, Deprez PH, Jenssen C (2017). Indications, results, and clinical impact of endoscopic ultrasound (EUS)-guided sampling in gastroenterology: European Society of Gastrointestinal Endoscopy (ESGE) Clinical Guideline - updated January 2017. Endoscopy.

[11] Bang JY, Hebert-Magee S, Trevino J, Ramesh J, Varadarajulu S (2012). Randomized trial comparing the 22-gauge aspiration and 22-gauge biopsy needles for EUS-guided sampling of solid pancreatic mass lesions. Gastrointest Endosc.

[12] Turner BG, Cizginer S, Agarwal D, Yang J, Pitman MB, Brugge WR (2010). Diagnosis of pancreatic neoplasia with EUS and FNA: a report of accuracy. Gastrointest Endosc.

[13] Siddiqui AA, Brown LJ, Hong SK (2011). Relationship of pancreatic mass size and diagnostic yield of endoscopic ultrasound-guided fine needle aspiration. Dig Dis Sci.

[14] Layfield LJ, Cramer H, Madden J, Gopez EV, Liu K (2001). Atypical squamous epithelium in cytologic specimens from the pancreas: cytological differential diagnosis and clinical implications. Diagn Cytopathol.

[15] Zacharia G, Levine J, Winstead NS, Antillon MR, Davis NK (2012). Primary squamous cell carcinoma of the pancreas diagnosed by endoscopic ultrasound-guided fine needle aspiration. J Gastrointestin Liver Dis.

[16] Armellini E, Manfrin E, Trisolini E (2019). Histologic retrieval rate of a newly designed side-bevelled 20G needle for EUS-guided tissue acquisition of solid pancreatic lesions. United European Gastroenterol J.

